# Modern Utility of Pelvimetry: a Relevant Tool or an Outdated Concept?

**DOI:** 10.7759/cureus.95522

**Published:** 2025-10-27

**Authors:** Margaret R Vitale, Daniel Shtirmer, Bryan P Ashley, Austin C Brown, Sean P Verrier, Stany Lobo

**Affiliations:** 1 Women's Health/Anatomical Sciences, Liberty University College of Osteopathic Medicine, Lynchburg, USA; 2 Anatomical Sciences, Liberty University College of Osteopathic Medicine, Lynchburg, USA; 3 Anatomy and Neurosciences, Liberty University College of Osteopathic Medicine, Lynchburg, USA

**Keywords:** cephalopevic disproportion, childbirth, dystocia, gynecology, labor, obstetrics, pelvimetry, pelvis

## Abstract

The use of clinical pelvimetry to assess the adequacy of the pelvis for vaginal birth is a controversial subject in modern-day obstetric and gynecological practice. Although it is not considered standard clinical practice in the United States, there is still an emphasis on teaching the concept as part of medical education and within clinical anatomy and obstetrics textbooks. We compiled research studies from the last decade to explore if pelvimetry is clinically useful and question its perceived importance in medical education.

## Introduction and background

Cephalopelvic disproportion (CPD) is a condition in which the fetus is unable to descend through the pelvis during normal vaginal labor, either because of fetal-pelvic fit (absolute) or fetal presentation and malposition (relative) [1]. The rate of CPD in the United States is thought to be one in 250 live births (0.4%) [2]. Pelvimetry is the medical practice of acquiring measurements of the female pelvis to identify potential cases of CPD; however, the clinical utility of these pelvimetric findings is being questioned in modern obstetric practice [3]. Clinical pelvimetry, as assessed by manual measurements, is included in current medical school curricula as well as in the clinical anatomy and obstetric textbooks used as primary texts for medical and postgraduate education. In clinical practice, pelvimetry is not regularly performed. The timing of when to perform pelvimetry is often a point of contention due to the significant changes the female body undergoes throughout the course of pregnancy [4]. The modern-day use of pelvimetry to predict CPD is a debated topic due to differing opinions on the accuracy of the measurements as well as the validity of using these measurements to determine a clinical course. This review uses relevant literature to examine whether the practice of pelvimetry provides value in clinical decision-making as well as whether its inclusion in medical school curricula and medical textbooks as standard practice should be reevaluated. 

History, pelvic anatomy, and pelvimetry methodology

When discussing pelvimetry, knowledge of the anatomical landmarks used in measurements provides the necessary context. The anteroposterior (AP) diameter is the distance between the pubic symphysis and the sacrococcygeal joint. The interspinous distance is the distance between the ischial spines and is the narrowest space through which the head of the fetus will pass. The transverse diameter is the distance between the ischial tuberosities. The obstetrical (true) conjugate spans from the middle of the sacral promontory to the posterosuperior margin of the pubic symphysis. The diagonal conjugate measures the distance of the sacral promontory to the inferior portion of the pubic symphysis [5,6]. 

To perform a manual internal pelvimetry assessment, the physician first measures the diagonal conjugate by inserting their hand into the vagina, palpating the sacral promontory with their middle finger, and marking their hand at the inferior margin of the pubic symphysis, measuring from the mark to the middle finger. The obstetrical (true) conjugate is then estimated by subtracting 1.5 cm (approximate difference between the middle and index finger) from the diagonal conjugate [5,6]. The curvature of the anterior surface of the sacrum is palpated to assess whether it is concave, flat, or convex. A flat or convex sacrum may indicate potential anterior-posterior constriction. The pelvic outlet is assessed by the clinician placing a fist between the ischial tuberosities, with a transverse diameter of 8.5 cm being considered adequate. The infrapubic angle is measured by positioning a thumb on each inferior pubic ramus and estimating the angle at which they meet, with an angle greater than 90 degrees considered ideal. The mid-pelvis cannot be measured with clinical examination but can be estimated by assessing the positioning of the pelvic sidewalls through the estimation of the ischial spines and sacrospinous ligament. This is estimated by first assessing the bispinous diameter of the ischial spines via palpation. The length of the sacrospinous ligament is estimated by palpating the ischial spine and the midline of the sacrum, with two-and-a-half finger lengths being considered adequate [4]. 

The Caldwell-Moloy classification system is the method most utilized to assess the shape of the pelvis. Using the measurements above, a clinician can identify a female’s pelvis as one of four shapes: gynecoid, anthropoid, android, and platypelloid (Table 1) [4-7].

**Table 1:** Caldwell-Moloy classification of pelvic shape The table was created by the authors using information synthesized from sources [4-7]. AP: anteroposterior

Caldwell-Moloy pelvic shape	Description
Gynecoid	Circular/ovular-shaped pelvis with a wide interspinous distance. Occurs in approximately 50% of women and is considered the most ideal shape for vaginal delivery.
Anthropoid	Ovoid-shaped pelvis with a long AP diameter. Occurs in approximately 20% of women and is considered appropriate for vaginal delivery.
Android	Small, heart-shaped pelvic inlet with a short AP and interspinous diameters. Occurs in less than 30% of women and is not considered suitable for vaginal delivery.
Platypelloid	Flattened gynecoid pelvis with a short AP diameter. Occurs in 3% of women. Vaginal delivery without operative intervention is unlikely to be successful.

Although the Caldwell-Moloy classification system is widely accepted, it is important to note that the studies from which the Caldwell-Moloy system was developed were conducted in the early 1900s. The first study used skeletonized bony pelvises to evaluate differences in shape and did not include information on whether the women had experienced difficult births. The second study analyzed radiographs only of women with a history of difficult childbirth experiences. These two studies lacked a control group, possibly skewing the results due to confounding variables and data bias. Additionally, the studies did not take into consideration the dynamic nature of the female pelvis in pregnancy, which is able to shift and stretch during childbirth due to the hormones induced by pregnancy and labor [8-11]. These study design flaws call into question the current utility and accuracy of the Caldwell-Moloy classification system and indicate that these measurements may be outdated. 

In modern-day medicine, pelvimetric measurements can be obtained by manual pelvic exam, X-ray, CT scan, and MRI. Imaging studies can be used to obtain more precise pelvimetric measurements, although the associated cost of these studies may place a financial burden on some patients [12]. Additionally, X-ray and CT scans should be avoided during pregnancy if they are not clinically necessary due to the risks of exposure to ionizing radiation [13]. Pelvimetric measurements, whether by imaging or manual exam, are often only assessed in one position and without specific guidance on when during pregnancy these measurements should be recorded. However, a 2021 study by Kjeldsen LL et al. used MRI technology to show that overall pelvic capacity increases between the second and third trimester. This study also showed that the sizes of the pelvic inlet and outlet were dependent on maternal position, with supine positioning increasing pelvic inlet size and semi-lithotomy and kneeling squat increasing pelvic outlet size. It was thought that this increased pelvic joint laxity occurs as a normal physiological change of pregnancy during the third trimester and introduces a margin of error into studies that assess pelvimetric measurements outside of the third trimester or in only one position [8]. 

Cesarean deliveries and CPD

The rate of cesarean section (C-section) has risen almost 12% since 1996. According to the Centers for Disease Control and Prevention (CDC), the United States had a C-section delivery rate of 32.3% in 2023. While statistical data categorizing the indication for C-section was not included in this report, 26.6% were considered low risk C-section deliveries, defined as C-sections performed on nulliparous women with singleton, cephalic presenting fetus at term (gestational age of 37 weeks or greater), and 8.5% of women who attempted a vaginal delivery with a vertex presenting fetus resulted in a C-section after a trial of labor [14]. However, it is important to note that an inadequate pelvis, as classified by the Caldwell-Moloy system, is not the only, or even the most common, precipitating factor of CPD. Fetal size, head circumference, and positioning are often contributing factors to cases of CPD [1]. Additionally, a retrospective study that examined vaginal birth after cesarean (VBAC) deliveries showed that among the women whose primary C-section was classified as being due to CPD, more than 60% went on to have a successful VBAC [15].

Dissent between medical textbooks

There is dissent between medical textbooks as to the relevance and importance of pelvimetric measurements. Some textbooks claim that clinical pelvimetry is a useful and relevant obstetric tool, whereas others claim that it is outdated and should not be considered as part of routine obstetric care. Anatomical textbooks tend to skew toward focusing on the maternal bony pelvis and consideration of pelvic shapes, as listed previously, as important aspects in predicting CPD. Gray’s Basic Anatomy, 3rd Edition states that “accurate measurements of a woman’s pelvic inlet and outlet can help in predicting the likelihood of a successful vaginal delivery during childbirth” [16]. Netter’s Essential Systems-Based Anatomy concludes, “not all women have a gynecoid pelvis; thus, pelvic measurements during pregnancy are important to assess the adequacy of the pelvis for childbirth” [17]. Hacker & Moore’s Essential Obstetrics and Gynecology definitively states that “clinical pelvimetry should be performed sometime before labor begins” and claims that this examination is “helpful for predicting potential problems during labor.” The authors state that pelvimetric measurements can be measured as early as the first prenatal visit, although they suggest that some obstetricians prefer to wait until later in pregnancy due to the physiological changes to the soft tissues and ligaments as the maternal body prepares for labor [4]. 

In contrast, Munro Kerr’s Operative Obstetrics, 13th Edition, outright states that “pelvimetry is not useful in deciding whether a pelvis is adequate, unless there is a history of bony disease or trauma” [18]. Clinical Obstetrics and Gynaecology, 5th Edition, makes no mention of clinical pelvimetry but references that pelvic deformation in high resource countries are rare, and that while imaging techniques such as X-ray, CT, and MRI can be used to determine pelvic measurements, they declare that these studies have “limited clinical value in predicting the likelihood of a successful vaginal delivery” [19]. Chestnut’s Obstetric Anesthesia echoes this assessment, stating that radiographic pelvimetry has “only a limited place in clinical management because of its poor ability to predict a successful vaginal birth,” and that due to numerous other factors that contribute to CPD, pelvimetric measurements alone will not accurately predict the presence or absence of CPD [20]. 

Interestingly, Gabbe's Obstetrics: Normal and Problem Pregnancies, 9th Edition, references clinical pelvimetry multiple times within different contexts. In Chapter 5, this textbook recommends that clinical pelvimetric measurements should be performed as part of standard prenatal care and should be measured at 6-8 weeks or the first prenatal visit [21]. This textbook then later claims in Chapter 13 that while determination of pelvic shape and capacity is still clinically useful, they also admit that it is a “very inexact science” and that trial of labor is the only definitive method of determination as to whether or not the mother and fetus will undergo a successful vaginal delivery [1]. 

## Review

Methods

As discussed, the indication for pelvimetric measurements is to assess whether a mother is suitable for a trial of vaginal delivery or if she requires an operative route. Pelvimetry, however, is an oversimplification of the factors that contribute to a difficult birth that may result in fetal distress, maternal morbidity, or the need for surgical intervention. In congruence with this oversimplification, the evidence exploring the validity of using pelvimetry to determine the safest method of delivery is sparse and controversial. For this analysis, we searched for articles written and published on PubMed, within the National Institutes of Health (NIH)’s National Library of Medicine, containing the words “Pelvimetry” and “Obstetrics” between January 2015 and January 2025 to search for research studies that provided evidence-based assessments of clinical pelvimetry. This search provided 67 results. Forty-two of these studies were not considered for review as they were not clinical studies with the direct goal of assessing the utility of pelvimetric measurements as a method of predicting successful vaginal births; an additional 11 studies were excluded as they focused solely on assessing the utility of pelvimetric measurements within the context of vaginal delivery of breech presentations; one study was excluded as it only assessed the utility of pelvimetry in short-statured women. Thirteen studies assessed the utilization of pelvimetric studies to assess their utility in the prediction of a successful vaginal birth and were included for review. 

Results

Two studies directly assessed the utility of clinical pelvimetric measurements. Neri S et al. found a significant inverse correlation between bituberous diameter (BTD) and unplanned operative interventions due to labor dystocia in low-risk nulliparous women, with an optimal cutoff value of 8.6 cm. In this study, the distance between the ischial tuberosities was assessed in lithotomic position via tape measure at 37-38 weeks of gestation. This study found a correlation between smaller BTD and longer first and second stages of labor but did not assess the quality of the maternal contractions, strength of uterine contractions, occipital position of the fetus, or position of the mother during labor, which are important factors in the progression of labor [22]. Roux N et al. assessed the value of pelvimetric measurements in patients attempting a trial of labor after cesarean section (TOLAC). While this study did find a higher rate of C-section in patients with abnormal pelvimetric findings (35.3% vs. 15.2%), the indication for C-section was not statistically significant between the two groups. It is important to note that knowledge of the abnormal pelvimetric findings was known to the obstetric care team, which presents a potential avenue for bias in the provider’s decision to intervene. Most notably, there was no significant difference in neonatal morbidity associated with abnormal pelvimetric dimensions in women who attempted a vaginal birth after C-section [23]. 

The two studies assessing the utility of X-ray pelvimetry (Komatsu M et al. and Pattinson RC et al.) both found that there was insufficient evidence to support the ability of X-ray pelvimetric measurements to predict CPD or the mode of delivery [3,24]. Komatsu M et al., which assessed pelvic size in the context of TOLAC deliveries, found that there were no statistically significant X-ray pelvimetric parameters associated with the success of TOLAC deliveries [24]. Pattinson RC et al., which assessed five previous trials and compared the results of women who underwent X-ray pelvimetry to either manual pelvimetry or no pelvimetry in cephalic presenting fetuses, similarly, did not find significant evidence in support of the predictive capabilities of X-ray pelvimetry in identifying cases of CPD. This study identified an increased likelihood for delivery via C-section after X-ray pelvimetry was performed and hypothesized that this dichotomy between the number of cesarean deliveries expected and performed was likely a result of bias (Table 2) [3].

**Table 2:** Pelvimetry studies - manual and X-ray ^a^ Arrest of labor, failure of induced labor. ^b^ Indication for C-section was not specified in this clinical review. The patients who had undergone X-ray pelvimetry had a higher risk of delivering via C-section than patients who had not undergone X-ray pelvimetry (520 per 1000 vs. 388 per 1000), with no significant difference in neonatal or maternal outcomes. ^c^ About 181 of these C-sections were in patients with abnormal pelvic dimensions (59% of total C-sections in this subgroup); 130 occurred in patients with normal pelvic dimensions (53% of total C-sections in this subgroup). ^d ^Reported C-sections and operative vaginal deliveries together. ^e^ Mode of delivery was statistically significant between normal and abnormal pelvic dimension groups, with C-sections being higher in the abnormal pelvic dimensions group; however, there were no statistically significant values for any indication between the two groups. ^f^ FHR abnormalities were more commonly the indication for operative vaginal deliveries than default of progression in patients with abnormal pelvic dimensions (142 (74%) vs. 50 (26%)); in patients with normal pelvic dimensions, FHR abnormalities accounted for the indication in 260 cases (57%) and default of progression in 201 cases (43%). FHR: fetal heart rate: C-section: cesarean section; CPD: cephalopelvic disproportion

Study name	Authors	Study year	Pelvimetry method	Study population size	Number of C-sections with obstetrical indications/suspected cephalopelvic disproportion^a^	Statistically significant measures	Pelvimetric measurements assessed during pregnancy	Pelvimetric measurements assessed during the 3^rd^ trimester	Fetal characteristics included in the CPD assessment	Obstetric team blinded to pelvimetric measurements	Pelvis measured in more than one position
Pelvimetry for fetal cephalic presentations at or near term for deciding on mode of delivery	Pattinson RC et al. [3]	2017	X-ray vs. manual or none	1159	Not specified^b^	1. Assessment of X-ray pelvimetric measurements	Not specified	Not specified	No	No	No
Trial of labor after cesarean and contribution of pelvimetry in the prognosis of neonatal morbidity	Roux N et al. [23]	2020	Manual	2474	311 (2.5%)^c^	1. Mode of delivery^e^	Not specified	Not specified	Yes	No	No
						2. Indication for obstetrical vaginal deliveries^f^					
Correlation between bituberous diameter and mode of delivery in a cohort of low-risk nulliparous women	Neri S et al. [22]	2023	Manual	116	7 (6%)	1. Bituberous diameter	Yes	Yes	Yes	Yes	No
						2. Length of the second stage of labor					
X-ray pelvimetry has no impact on the outcomes of trial of labor after cesarean delivery: a retrospective single-center study	Komatsu M et al. [24]	2024	X-ray	96	8 (8.3%)^d^	1. Previous C-section due to failed trial of labor	Yes	Yes	Yes	Yes	No
						2. Newborn’s weight at birth					

Eight studies assessed the use of either MRI or CT imaging to obtain pelvimetric measurements and assess the ability to predict cases of CPD. Although many of these studies assessed the same anatomical landmarks, there was very little overlap in results. Most studies identified different pelvic components as being statistically significant indicators of CPD, with some not finding any statistically significant measures at all. Others only found significance when the pelvic measurements were considered within the context of fetal dimensions, although this was not always the case either. Chen C et al. found that fetal head circumference and fetal abdominal circumference had the greatest statistically significant effect on predicting CPD and recommended using a multifactorial approach to assessing the risk of CPD with consideration of both maternal and fetal measurements [25]. Ami O et al. calculated the potential percentage of fetal head compression as a result of cephalopelvic fit to try to identify potential cases of CPD resulting in C-section and hypothesized that a reduction in emergency C-sections could be achieved using this model, although statistical significance between groups was not overtly reported [26]. Li YG et al., Li J et al., and Liberty G et al. all found that the statistically significant relationships with the best potential clinical significance occurred when calculating cephalopelvic fit. Multiple studies noted that although they were able to calculate statistically significant results, this did not immediately indicate clinically significant findings [27-29]. Korhonen U et al. specifically determined that while consideration of the fetal-pelvic index (FPI) at higher values was associated with a greater odds of C-sections, the area under the curve (AUC) value of the receiver operating characteristic (ROC) curve showed that the FPI was a poor predictor of CPD resulting in C-sections [30]. Comparatively, Liao KD et al. and Starrach T et al. did not consider fetal dimensions within their prediction of CPD [31,32]. Liao KD et al. found that although pelvic dimensions were larger on average for women who had successful vaginal deliveries compared to cases of CPD, there was also significant overlap of the pelvic sizes between both groups of women [31]. In the study performed by Starrach T et al., the pelvic inlet was found to be 7% lower for C-sections compared to vaginal delivery; however, this study used data gathered in women aged 50 and older, did not consider fetal factors, and acknowledged significant overlap between their study groups (Table 3) [32].

**Table 3:** Pelvimetry studies - CT and MRI ^a^ Arrest of labor, failure of induced labor. ^b^ Indication for C-section not reported. ^c^ A total of 19/104 CT scans were taken during pregnancy, gestational age not reported. ^d^ Noted by the article that while statistically significant, ROC values, sensitivity, and specificity were small/not clinically significant. ^e^ Specifically noted that fetal-pelvis index was not statistically significant in predicting arrest of labor due to CPD. ^f ^While this study showed that the STOL score was positively correlated with a higher rate of C-sections related to CPD, it did not include specific p-values between different measures to prove statistical significance. Additionally, there appears to be significant overlap between confidence intervals of the majority of measures reported, further calling into question the significance of this data. ^g^ Cases were matched with controls of similar birthweights; however, fetal characteristics were not considered within statistical calculations. CPD: cephalopelvic disproportion; CPCI: cephalopelvic circumference index; C-section: cesarean section; ROC: receiver operating characteristic; STOL: simulated trial of labor; BPD: biparietal diameter; N/A: not applicable

Study name	Authors	Study year	Pelvimetry method	Study population size	Number of C-sections with obstetrical indications/suspected cephalopelvic disproportion^a^	Statistically significant measures	Pelvimetric measurements assessed during pregnancy	Pelvimetric measurements assessed during the 3^rd^ trimester	Fetal characteristics included in the CPD assessment	Obstetric team blinded to pelvimetric measurements	Pelvis measured in more than one position
Fetal pelvic index to predict cephalopelvic disproportion - a retrospective clinical cohort study	Korhonen U et al. [30]	2015	MRI	274	32 (11.7%)	None^e^	Yes	Yes	Yes	No	No
			X-ray								
Study on the cephalopelvic relationship with cephalic presentation in nulliparous full-term Chinese pregnant women by MRI with three-dimensional reconstruction	Li YG et al. [27]	2018	MRI	301	32 (10.6%)^b^	1. Neonatal weight	Yes	Yes	Yes	No	No
						2. BMI					
						3. Transverse diameter of pelvic inlet^d^					
						4. Posterior sagittal diameter of mid-pelvis^d^					
						5. Fetal BPD					
						6. Fetal occipitofrontal diameter					
						7. Fetal suboccipitobregmatic diameter					
						8. Fetal occipitomental diameter					
						9. Difference between minimum mid-pelvic diameter and BPD					
						10. Difference between mid-pelvic circumference and fetal head circumference					
						11. Difference between mid-pelvic area and fetal head area					
						12. Difference between mid-pelvic volume and fetal head volume					
Three-dimensional magnetic resonance pelvimetry: a new technique for evaluating the female pelvis in pregnancy	Liao KD et al. [31]	2018	MRI	223	13 (5.8%)	1. Post-sagittal diameter of pelvic outlet	Yes	Yes	No^g^	Yes	No
						2. Transverse diameter of pelvic inlet					
						3. Anterior-posterior diameter of the mid-pelvis					
						4. Anterior-posterior diameter of the pelvic outlet					
The relation between head circumference and mid-pelvic circumference: a simple index for cephalopelvic disproportion evaluation	Liberty G et al. [29]	2021	CT	104	20 (19.2%)	1. Transverse diameter of the mid-pelvis	No^b^	N/A	Yes	Unknown	No
						2. Pelvic circumference of the mid-pelvis					
						3. Neonatal head circumference					
						4. CPCI of the pelvic inlet					
						5. CPCI of the mid-pelvis					
						6. CPCI of the pelvic outlet					
Magnetic resonance imaging-based nomogram to antenatal predict cesarean delivery for cephalopelvic disproportion in primiparous women	Chen C et al. [25]	2022	MRI	150	61 (40.7%)	1. BMI	Yes	Yes	Yes	Yes	No
						2. Fetal head circumference					
						3. Fetal abdominal circumference					
						4. Maternal bilateral femoral head distance					
						5. Obstetric conjugate					
Childbirth simulation to assess cephalopelvic disproportion and chances for failed labor in a French population	Ami O et al. [26]	2023	MRI	314	78 (24.8%)	None^f^	Yes	Yes	Yes	No	No
Predictive value of MRI pelvimetry in vaginal delivery and its practicability in prolonged labour - a prospective cohort study	Li J et al. [28]	2023	MRI	150	25 (16.7%)	1. Fetal head circumference	Yes	Yes	Yes	Yes	No
						2. Cephalopelvic index of diameter					
						3. BMI					
						4. Fetal abdominal circumference					
						5. Interspinous diameter					
						6. Intertuberous diameter					
Pelvic inlet area is associated with birth mode	Starrach T et al. [32]	2023	CT	139	21 (15.1%)^b,c^	1. Pelvic inlet circumference	No	N/A	No	Unknown	No

The final study assessing the relationship between pelvimetry and CPD is part of a novel research project by Siccardi M et al., assessing the relationship between external anatomical landmarks and the anatomical mobility of the pelvis with obstetric outcomes. Siccardi M et al. used palpable bony landmarks to calculate how the dimensions of the pelvis change in relation to maternal positioning and the relationship between these measurements with cases of CPD. While this project offers a significantly less invasive and more affordable method of assessing the maternal pelvis as well as integrating the positional dynamics of the pelvis, this study did not include fetal dimensions as part of the evaluation of CPD (Table 4) [33].

**Table 4:** Pelvimetry studies - other ^a^ Arrest of labor, failure of induced labor. DEP: dynamic external pelvimetry; C-section: cesarean section; CPD: cephalopelvic disproportion

Study name	Authors	Study year	Pelvimetry method	Study population size	Number of C-sections with obstetrical indications/suspected cephalopelvic disproportion^a^	Statistically significant measures	Pelvimetric measurements assessed during pregnancy	Pelvimetric measurements assessed during the 3^rd^ trimester	Fetal characteristics included in the CPD assessment	Obstetric team blinded to pelvimetric measurements	Pelvis measured in more than one position
Can the dynamic external pelvimetry test in late pregnancy reveal obstructed and prolonged labor? Results from a pilot study	Siccardi M et al. [33]	2021	DEP	70	9 (11%)	1. Longitudinal hemi-diameter of the sacral area of Michaelis in straight legs, hands-and-knees, and kneeling squat positions	Yes	Yes	Yes	No	Yes
						2. Inter-tuberous diameter in straight legs, hands-and-knees, and kneeling squat positions					
						3. Base of Trillat’s triangle, in straight legs, hands-and-knees, and kneeling squat positions					
						4. Difference in transverse diameter of the sacral area of Michaelis between straight legs and hands-and-knees positions					
						5. Difference in sizes of the bi-cristal diameter between hands-and-knees and kneeling squat positions					

Discussion

The use of manual estimations of pelvimetric measurements, as it is frequently discussed in textbooks and medical education, does not appropriately reflect the current clinical utility of these measurements. There are very few studies performed in the last decade that assessed the accuracy or utility of pelvimetric measurements in predicting successful cephalic vaginal deliveries, with a high majority using imaging studies rather than manual measurements. A majority of studies included fetal dimensions and cephalopelvic fit as part of their screening for CPD, which is a departure from the textbook standard of unilaterally using pelvic measurements. While most studies were able to report at least one statistically significant finding, very few studies found the same measure to be statistically significant, indicating that these results do not appear to be consistently reproducible and have little clinical significance. A majority of studies also had very small sample sizes and case numbers, with only two studies available that were able to include more than 100 cases of CPD. 

There is no modern evidence that a manual assessment of pelvimetry has any benefit to prenatal or intrapartum care. It was noted that the use of pelvimetry increases the frequency of electing to perform a C-section [3]. As only five of the 13 studies reviewed published definitively blinded results, this introduces the possibility of unintentional bias toward a more conservative approach during labor. While one of the main arguments for assessing cephalopelvic adequacy is to decrease the rate of unscheduled C-sections due to concern of increased adverse outcomes, Roux N et al. showed that there was no increase in neonatal morbidity regardless of cephalopelvic fit [23].

The large majority of studies also only assessed pelvimetric measurements in a single, static position. As Kjeldsen LL et al. and Siccardi M et al. have discussed, maternal positioning may significantly impact pelvic adequacy. This avenue should continue to be explored as a potential method to reduce unplanned C-section deliveries as a result of CPD [8,9].

## Conclusions

One of the golden rules of medicine is that a test should only be ordered if the results will significantly change the plan of care, and with clinical pelvimetry, it has been repeatedly determined that while these measurements may provide some ability to predict CPD, the only true test of pelvic adequacy is by a trial of labor. Due to the lack of evidence supporting the utility of pelvimetry as a screening tool for CPD, potential for bias, additional costs, and use of resources, we do not recommend the use of pelvimetry within standard obstetric care for patients. 

Imaging studies to assess the pelvis may be useful on a case-by-case basis, such as for women with a history of pelvic abnormalities, trauma, or who are at risk for difficult vaginal deliveries due to fetal presentations, although this was not the focus of our literature analysis. The safety and comfort of both the mother and the fetus are of utmost importance in obstetric consideration. As the research indicates, pelvimetry should not serve as a factor in clinical decision-making for most cases. The clinical and educational emphasis it currently receives should be amended to better align with modern-day obstetric practice, with reference only to the historical significance of clinical pelvimetry as a standard of care. 
